# Histopathological Analysis of Adrenal Glands after Simian Varicella Virus Infection

**DOI:** 10.3390/v13071245

**Published:** 2021-06-26

**Authors:** Christy S. Niemeyer, Teresa Mescher, Rocio Griggs, David J. Orlicky, Gregory K. Wilkerson, Andrew N. Bubak, James E. Hassell, Brittany Feia, Ravi Mahalingam, Vicki Traina-Dorge, Maria A. Nagel

**Affiliations:** 1 Department of Neurology, University of Colorado School of Medicine, Aurora, CO 80045, USA; christy.niemeyer@cuanschutz.edu (C.S.N.); teresa.mescher@cuanschutz.edu (T.M.); rocio.griggs@cuanschutz.edu (R.G.); andrew.bubak@cuanschutz.edu (A.N.B.); james.hasselljr@cuanschutz.edu (J.E.H.J.); brittany.feia@cuanschutz.edu (B.F.); ravi.mahalingam@cuanschutz.edu (R.M.); 2 Department of Pathology, University of Colorado School of Medicine, Aurora, CO 80045, USA; david.orlicky@cuanschutz.edu; 3 Department of Comparative Medicine, Keeling Center for Comparative Medicine and Research, University of Texas, MD Anderson Cancer Center, Bastrop, TX 78602, USA; gkwilkerson@mdanderson.org; 4 Microbiology Division, Tulane National Primate Research Center, Tulane University, Covington, LA 70433, USA; vtraina@tulane.edu; 5 Department of Ophthalmology, University of Colorado School of Medicine, Aurora, CO 80045, USA

**Keywords:** varicella zoster virus, simian varicella virus, adrenal glands, adrenal dysfunction, hypothalamic-pituitary-adrenal axis

## Abstract

Latent varicella zoster virus (VZV) has been detected in human adrenal glands, raising the possibility of virus-induced adrenal damage and dysfunction during primary infection or reactivation. Rare cases of bilateral adrenal hemorrhage and insufficiency associated with VZV reactivation have been reported. Since there is no animal model for VZV infection of adrenal glands, we obtained adrenal glands from two non-human primates (NHPs) that spontaneously developed varicella from primary simian varicella virus (SVV) infection, the NHP VZV homolog. Histological and immunohistochemical analysis revealed SVV antigen and DNA in the adrenal medulla and cortex of both animals. Adrenal glands were observed to have Cowdry A inclusion bodies, cellular necrosis, multiple areas of hemorrhage, and varying amounts of polymorphonuclear cells. No specific association of SVV antigen with βIII-tubulin-positive nerve fibers was found. Overall, we found that SVV can productively infect NHP adrenal glands, and is associated with inflammation, hemorrhage, and cell death. These findings suggest that further studies are warranted to examine the contribution of VZV infection to human adrenal disease. This study also suggests that VZV infection may present itself as acute adrenal dysfunction with “long-hauler” symptoms of fatigue, weakness, myalgias/arthralgias, and hypotension.

## 1. Introduction

Varicella zoster virus (VZV; human herpesvirus 3) is an exclusively human, double-stranded DNA virus that infects >90% of the world population. Primary infection occurs after exposure to aerosolized virus or vesicular fluid from individuals with varicella (chicken pox) or herpes zoster (shingles). VZV replicates in the upper respiratory mucosal epithelial cells, spreads to tonsils and other lymphoid tissue, and infects T cells. Infected T cells then enter the bloodstream and transport VZV to the skin, causing a widespread rash (varicella) (reviewed in [1]). The virus enters sensory neurons of the cranial, dorsal root, autonomic, and enteric ganglia to establish latency through infected T cells or by retrograde transport of virions from infected skin [2,3,4,5,6]. With aging or immunosuppression, VZV reactivates from one or more ganglia and typically travels to the skin to produce herpes zoster in the corresponding dermatome. VZV can also spread to other organs innervated by infected neurons and cause disease corresponding to sites of virus replication and inflammation with or without rash.

**Figure 1:**
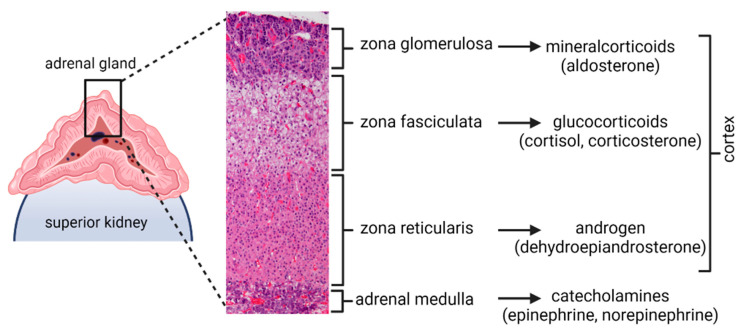
Adrenal gland schematic adjacent to a normal macaque adrenal gland stained by hematoxylin and eosin. Illustrations adapted from Biorender.com (accessed on 25 June 2021).

The adrenal gland is an important organ that can be affected by pathogens. The adrenal glands are part of the hypothalamic-pituitary-adrenal (HPA) axis, a major neuroendocrine system that mediates the body’s response to stress and regulates multiple processes, including digestion, immunity, and mood [7,8]. The adrenal glands are located adjacent to the superior pole of each kidney and are composed of two distinct anatomical regions, the cortex and the medulla (Figure 1). The cortex itself comprises three zones, with each zone secreting specific hormones: (1) the zona glomerulosa (secretes mineralocorticoids, such as aldosterone, which regulates blood pressure and salt balance); (2) zona fasciculata (secretes glucocorticoids cortisol and cortisone, which regulates metabolism and immune system suppression); and (3) zona reticularis (secretes androgens; converted to sex hormones by gonads and other organs). The medulla primarily comprises chromaffin cells, which secrete catecholamines (epinephrine and norepinephrine). Since chromaffin cells within the adrenal medulla are neural crest-derived, like the dorsal root and sympathetic ganglionic neurons where VZV is latent, we previously examined 63 human adrenal glands obtained from human pheochromocytoma resections and kidney transplantation surgeries and found that four (6%) contained VZV DNA, but not VZV RNA or antigen, indicating that a virus may establish latency in adrenal glands [6]. These results raised questions about how a virus enters adrenal glands during primary infection and how a virus can cause adrenal dysfunction during primary infection or reactivation. Primary VZV infection has been associated with adrenal dysfunction and bilateral adrenal hemorrhage (Waterhouse–Friderichsen syndrome) in rare case reports [9,10,11,12]. Since there is no animal model for VZV infection of adrenal glands, we used the simian varicella virus (SVV) model of infection in non-human-primates (NHPs) to study pathologies associated with infected adrenal glands. SVV is an ideal animal model for VZV infection because it recapitulates human disease produced by VZV. SVV primary infection in NHPs causes varicella, after which a virus establishes latency in ganglionic neurons; immunosuppression triggers reactivation resulting in zoster [13,14,15,16,17,18]. In addition, SVV antigen has been detected in adrenal glands during primary infection and during latency in African green monkeys and rhesus macaques [14,19,20]. For this study, we obtained rare SVV-infected adrenal glands from two NHPs with naturally acquired varicella to further elucidate viral effects on adrenal glands through histopathological and molecular analysis.

## 2. Materials and Methods

### 2.1. Animal Infection and Tissue Collection

The first NHP was a 15-year-old, immunocompetent, SVV-seronegative female cynomolgus macaque (*Macaca fascicularis*; “immunocompetent”) that acquired primary SVV infection from a subclinical SVV-reactivating housing mate at the MD Anderson Cancer Center, Keeling Center for Comparative Medicine and Research (Bastrop, TX, USA). The animal developed a disseminated rash with SVV DNA detected in skin lesions and multiple organs by polymerase chain reaction (PCR). Humane euthanasia and necropsy were performed 1 day after rash onset. Formalin-fixed and paraffin-embedded (FFPE) adrenal gland blocks were prepared, and slide sections cut for analysis. The second NHP was a five-year-old, SVV-seronegative male rhesus macaque (*Macaca mulatta*; “irradiated”) that was treated with full-body gamma irradiation at the University of Maryland, School of Medicine (Baltimore, Maryland), and developed a disseminated rash 105 days later, which was confirmed to be varicella due to primary SVV infection, as previously described [19]. SVV was most likely acquired from a housing mate. Humane euthanasia and necropsy were performed six hours after rash onset; multiple tissues were collected and fixed, including adrenal glands, then slides sectioned. A control adrenal gland was commercially acquired from a five-year-old male, SVV-seronegative rhesus macaque (Primate Biologicals, Miami, FL, USA). All animal care guidelines were followed according to the respective institutions where animals were maintained.

### 2.2. Immunohistochemistry

FFPE adrenal glands were cut into 5 µm thick sections, baked onto slides at 60 °C for 1 h, and then deparaffinized. One slide from each of the two SVV-infected animals and one control animal was stained with hematoxylin and eosin (H&E) for histopathological analysis. Slides prepared from tissue sections adjacent to the H&E adrenal sections underwent antigen-retrieval in citrate buffer (pH = 6) for 10 min at 95 °C. These slides were dual immunostained using ImmPRESS staining kit (Vector Laboratories, Inc, Burlingame, CA) per manufacturer’s instructions using a rabbit anti-SVV 63 antibody (1:7000) [21] and one of the three following antibodies: (1) mouse anti-CD45 (1:100; Agilent Technologies Catalog No. M0701, Santa Clara, CA, USA) that detects leukocytes; (2) mouse anti-tyrosine hydroxylase (TH; 1:500; LSBio Catalog No. LS-B12072, Seattle, WA, USA) that detects epinephrine- and norepinephrine-secreting chromaffin cells in the adrenal medulla; or (3) mouse anti-βIII tubulin (1:500; StemCell Technologies Catalog No. 60052, Vancouver, BC, Canada) that detects nerve fibers. Archived FFPE samples from a macaque with disseminated SVV infection were used as positive controls for SVV antigen (duodenum), CD45 antigen (lymph node), TH antigen (adrenal gland), and βIII tubulin (dorsal root ganglia). For control sections, primary antibodies were replaced with appropriate dilutions of normal rabbit serum (NRS) [21] and mouse IgG1 isotype control antibody (MIgG1; Agilent Technologies). All stained slides were visualized and imaged using a BX46 microscope and CellSens software (Olympus, Center Valley, PA, USA).

### 2.3. DNA Extraction and Quantitative PCR

Three adrenal gland sections from each of the two SVV-infected animals (SVV antigen-positive regions) and one control (SVV antigen-negative regions) were scraped, DNA extracted, and analyzed by qPCR as described in [22] using primers for glyceraldehyde 3-phosphate dehydrogenase (GAPdH; FWD: CACATGGCCTCCAAGGAGTAA; REV: TGAGGGT- CTCTCTCTTCCTCTTGT; probe: VIC/CTGGACCACCAGC-CCCAGCAAG-/BkFQ/) and SVV open reading frame (ORF)-61 (forward primer 5′-ACACAGCGCTAATGAGAAGCC-3′; reverse primer 5′-GAAAGACGCTGCTGTTGTCG-3′; probe 5′-FAM/CAACCCCGCGTGTTGGCCC/3BHQ-3′). SVV DNA copy numbers were calculated and normalized to the quantity of DNA in the assay.

## 3. Results

### 3.1. Adrenal Glands from Macaques with Varicella Contain Cowdry A Inclusion Bodies, Polymorphonuclear Cells (PMNs), Necrotic Cells, and Hemorrhagic Regions

**Figure 2:**
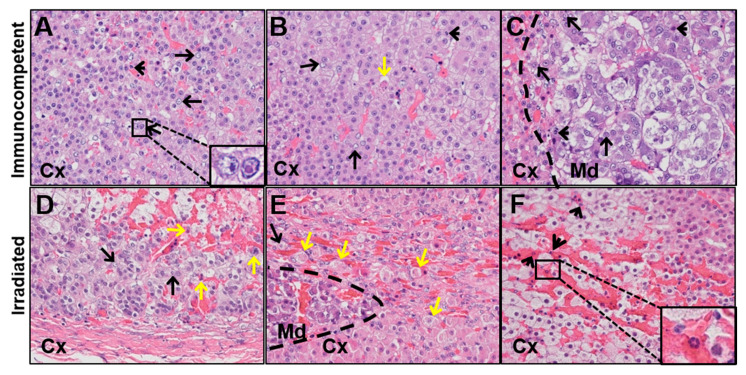
Histopathological abnormalities in adrenal glands of SVV-infected immunocompetent and irradiated macaques. An adrenal gland was obtained at necropsy from an immunocompetent macaque and an irradiated macaque with varicella due to primary SVV infection; adrenal glands were sectioned and stained with hematoxylin and eosin. (**A**,**B**) The adrenal cortex of the immunocompetent animal contained Cowdry A inclusions (long, black arrows), polymorphonuclear cells (PMNs) (short, black arrows), rare necrotic cells (yellow arrow), and hemorrhagic regions. Inset in (**A**) is a representative Cowdry A inclusion body on the right, adjacent to a normal nucleus on the left; (**C**) the adrenal medulla of the immunocompetent animal also contained Cowdry A inclusions and PMNs; (**D**,**E**) the adrenal cortex of the irradiated animal contained Cowdry A inclusions and multiple necrotic cells; none were seen in the medulla. More extensive hemorrhagic regions in cortex, extending to medulla, were present; (**F**) the adrenal cortex of the irradiated animal also contained PMNs (inset, high magnification). Magnification 100×, insets 400×. Crtx, cortex; Md, medulla; dashed line, corticomedullary junction.

An adrenal gland was obtained at necropsy from each of the two macaques with varicella (one immunocompetent and another irradiated) and stained with H&E. Histologic and pathologic findings from these sections are provided in Table 1. In the immunocompetent animal (Figure 2A–C), Cowdry A inclusion bodies (long, black arrows) and rare polymorphonuclear cells (PMNs; short, black arrows) were seen in the adrenal cortex and medulla. Rare necrotic/apoptotic cells were seen in the cortex (Figure 2B; yellow arrow) and diffuse mild hemorrhage was seen throughout. In the irradiated animal (Figure 2D–F), Cowdry A inclusions and rare PMNs were only found in the cortex. Compared to the immunocompetent animal’s adrenal gland, there were more necrotic cells in the cortex, and the hemorrhage was greater in severity throughout the cortex and medulla.

### 3.2. Adrenal Glands from Macaques with Varicella Contain SVV Antigen Yet Differ in CD45 Expression

**Figure 3:**
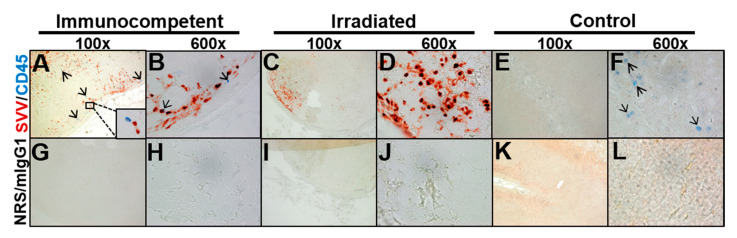
Immunohistochemical analysis for cells expressing SVV antigen and CD45 in adrenal glands from an immunocompetent and irradiated macaque with varicella. (**A**,**B**) In the adrenal gland from the immunocompetent macaque, SVV antigen-positive cells (red color) were detected in the adrenal cortex and medulla. CD45-positive cells (blue color and arrows) were also seen in the adrenal cortex and medulla. (**C**,**D**) In the adrenal gland from the irradiated macaque, SVV antigen-positive cells were detected in the cortex, but no CD45-positive cells were seen in either medulla or cortex. (**E**,**F**) In the control adrenal glands, no SVV antigen-positive cells were detected, and rare CD45-positive cells were seen. (**G**–**L**) No staining was seen when primary antibodies were replaced with normal rabbit serum (NRS) and mouse IgG1 isotype control antibodies. Magnification 100× (**A**,**C**,**E**,**G**,**I**,**K**) and 600× (**B**,**D**,**F**,**H**,**J**,**L**).

Sections of adrenal glands from the two macaques with varicella and the uninfected control animal were immunostained for the presence of SVV and CD45 antigens. Confirming the distribution of Cowdry A inclusions seen in H&E (Figure 2), cells expressing SVV antigen were detected in the adrenal cortex and medulla of the immunocompetent animal and only in the cortex of the irradiated animal (Figure 3A–D; red color); SVV antigen was not detected in the control adrenal gland from an uninfected macaque (Figure 3E,F). CD45-positive cells (blue color) were found in the adrenal cortex and medulla of the immunocompetent macaque (Figure 3A,B; black arrows), but not in the irradiated macaque (Figure 3C,D). Rare CD45-positive cells were seen in the medulla of the control adrenal gland (Figure 3E,F). No staining was seen when primary antibodies were replaced with NRS and mIgG1 control antibodies (Figure 3G–L). Adrenal gland sections that were positive for SVV antigen were scraped and confirmed to have SVV ORF-61 DNA by PCR (immunocompetent 104.5 copies/ng of DNA; irradiated: 16735.8 copies/ng DNA).

### 3.3. Adrenal Glands from Macaques with Varicella Have Varying Distributions of SVV Antigen in Cortex and Medulla without a Clear Association with Nerve Fibers

**Figure 4:**
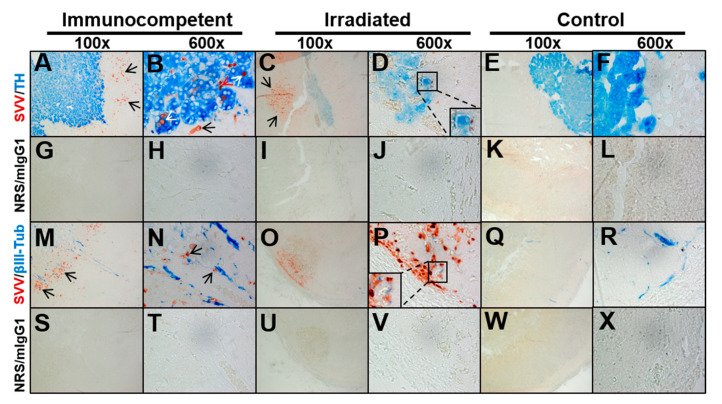
Immunohistochemical analysis to determine the location of simian varicella virus (SVV) in adrenal glands and association with nerve fibers. Adrenal gland slides from immunocompetent or irradiated macaque with varicella, as well as from a control uninfected macaque, were immunostained for the presence of SVV antigen, tyrosine hydroxylase (TH, marker for medullary chromaffin cells), and beta-3 tubulin (βIII tubulin, marker for nerve fibers) antigen. (**A**–**D**) SVV antigen-positive cells were detected in the cortex of infected adrenal gland of both animals (red color, black arrows); (**B**) In the immunocompetent animal, SVV antigen was seen in TH-negative cells within the cortex, as well as in TH-negative and TH-positive (blue) chromaffin cells within the medulla (red and white arrows, respectively). (**C**) In the irradiated animal, SVV antigen was seen in TH-negative cells in the cortex (black arrows). (**D**) Rare SVV antigen-positive cells were seen at the corticomedullary junction of the irradiated macaque (inset, 600× high magnification). (**E**,**F**) No SVV antigen-positive cells were detected in the control. (**M**–**P**) SVV antigen was once again detected in the infected adrenal glands of both infected animals (red color) and was located near βIII tubulin-positive nerves (blue color, arrow; 600× high-power magnification in inset (**P**)). (**Q**,**R**) No SVV antigen-positive cells were detected in the control adrenal gland; however, βIII tubulin-positive nerves were detected (blue color). (**G**–**L**,**S**–**X**) No staining was seen when primary antibodies were replaced with normal rabbit serum (NRS) and mouse IgG1 isotype control antibodies. Magnification 100× (**A**,**C**,**E**,**G**,**I**,**K**,**M**,**O**,**Q**,**S**,**U**,**W**) and 600× (**B**,**D**,**F**,**H**,**J**,**L**,**N**,**P**,**R**,**T**,**V**,**X**).

To determine the anatomical location of SVV antigen, adrenal gland sections from the immunocompetent and irradiated macaques with varicella and the control animal were immunostained for the presence of SVV and TH antigen to identify chromaffin cells (Figure 4A–F). SVV antigen was detected in the cortex of infected adrenal glands of both animals with varicella (Figure 4A–D; red color). In the immunocompetent animal, SVV antigen was detected in TH-negative cells within the cortex (Figure 4A,B; black arrows). Within the medulla, SVV antigen was detected in TH-positive chromaffin cells (white arrows) and TH-negative non-chromaffin cells (red arrow). In the irradiated animal, SVV antigen was not identified in the medulla itself but was detected in TH-negative cells in the cortex (Figure 4C; black arrows) and corticomedullary junction (Figure 4D; inset, high magnification). No SVV antigen was detected in the control adrenal gland (Figure 4E,F). No SVV antigen or TH were detected when primary antibodies were replaced with NRS and mIgG1 control antibodies (Figure 4G–L).

Alphaherpesviruses can spread to organs along nerve fibers. Therefore, to determine if SVV is associated with nerve fibers, adrenal glands were immunostained for the presence of SVV antigen and beta-3 tubulin (βIII tubulin, marker for nerve fibers) antigens. SVV antigen was once again detected in the infected adrenal cortex of both animals with varicella (Figure 4M–P; red color, black arrows) and was located near βIII tubulin-positive nerves (blue color; high-power magnification of SVV antigen adjacent to nerve fibers in inset P); however, a definitive association was difficult to establish. Interestingly, there was less βIII tubulin in the irradiated adrenal gland, most likely due to the extensive necrosis. No SVV antigen was detected in the control adrenal gland, although βIII tubulin-positive nerves were seen (Figure 4Q,R; blue color). No staining was seen when primary antibodies were replaced with NRS and mIgG1 control antibodies (Figure 4G–L,S–X).

## 4. Discussion

Herein, we examined the immunohistopathological characteristics of adrenal glands containing SVV antigen in two macaques with varicella. In the immunocompetent macaque, SVV antigen was detected within the cortex and medulla (in chromaffin and non-chromaffin cells), along with necrosis detected predominantly in the cortex and hemorrhage throughout. In the irradiated animal, SVV antigen was present in the cortex and corticomedullary junctions but absent in the medulla; more hemorrhage and necrosis were seen throughout. The increased cell death and hemorrhage, as well as an absence of CD45-positive cells in the adrenal gland of the irradiated animal, is not surprising as radiation therapy is known to cause necrosis, thrombocytopenia, and leukopenia [23].

Histopathological characterization of adrenal glands from macaques acutely infected with SVV sheds light on the biological basis of clinical manifestations associated with varicella virus infection. More extensive histopathological studies on adrenal glands have been reported for the closely related herpes simplex virus (HSV). Specifically, Potratz et al. [24] infected mice intraperitoneally with several strains of HSV (HSV-1 and HSV-2) and found morphological alterations in the zona fasciculata, including necrosis. HSV antigen was detected but cleared 10 days after inoculation, and the viral loads were strain-dependent. Compared to the paucity of CD45 cells in the SVV-infected adrenal glands of the irradiated animal, inflammatory infiltrates comprised of macrophages, granulocytes, and T helper cells (but only a few T-cytotoxic/suppressor lymphocytes) were present in HSV-1-infected adrenal glands. Similarly, mice infected intraperitoneally with neuroinvasive strains of HSV-1 and -2 had viral antigen in the adrenal cortex, primarily in regions corresponding to the human zona fasciculata and zona reticularis, extending into the adrenal medulla [25]. Investigators found HSV-1 antigens around necrotic regions but no evidence of HSV-1 latency. However, the same group, when infecting HSV-1 intravaginally, found HSV-1 antigens and Cowdry A inclusion bodies in the medulla but not in the cortex, suggesting that the localization of HSV-1 infection to specific adrenal regions depends on the route of inoculation [26]. Finally, Nakamura et al. [27] studied adrenal glands obtained from an autopsy of six human neonates with HSV infection. They found HSV-1 antigens in multiple organs, including the adrenal gland, in five out of the six cases, with extensive coagulative necrosis and Cowdry A inclusion bodies. Despite the morphological changes observed in HSV-1-infected neonatal adrenal glands, to our knowledge, there are no clear cases of HSV-1 infection in humans associated with adrenal insufficiency beyond a complex case report involving a 75-year-old woman with a urinary tract infection caused by *Escherichia coli*. She was diagnosed with iatrogenic tertiary adrenal insufficiency and subsequently developed a gastric ulcer due to HSV-1. Given her multiple infections, as well as steroid use, it is unclear whether HSV-1 caused adrenal dysfunction [28]. Rodent studies have shown disruptions in ACTH and corticosteroids during HSV infection; however, it is unclear whether they were a direct effect of HSV infection of adrenal glands or disruption along the HPA axis [29,30].

We could not find a clear association of SVV antigen with nerve fibers; thus, it is still unclear whether the virus entered adrenal glands via spreading to ganglionic neurons that innervate the gland or via hematogenous spread. However, studies of pseudorabies virus (PRV) have identified autonomic innervation of adrenal glands, supporting a potential route of infection to and from adrenal glands for other neurotropic viruses [31]. After injecting PRV into rat adrenal glands, the virus was detected in sympathetic preganglionic neurons and the brainstem, demonstrating that adrenal glands are innervated by sympathetic nerve fibers, and the virus can spread along these fibers to the CNS [32,33]. Field and Hill [34] injected PRV into the footpads of rats and found the virus in their adrenal glands; the infection was reduced following sympathectomy, suggesting that the route of adrenal gland infection from the periphery was along sympathetic fibers. Similarly, PRV can cause gross hemorrhage and necrosis in the adrenal glands of young pigs, with positive PRV antigens found within the medulla and immune cell infiltrates found in adrenal glands [35].

For both SVV-infected macaques studied herein, the preponderance of adrenal pathology was within the cortex. Given the observed cell death and hemorrhage in the cortex, one would expect a deficiency in the secretion of these hormones. Indeed, the presence of adrenal hemorrhage in SVV-infected adrenal glands recapitulates multiple case reports associated with VZV infection. Heitz et al. [12] described a 53-year-old man with abdominal pain whose CT scan revealed diffuse swelling and inflammation around both adrenal glands. He subsequently developed varicella, followed by bilateral adrenal hemorrhage (Waterhouse–Friderichsen syndrome), hypotension, salt imbalance, and hypercortisolism; despite antiviral therapy, hypercortisolism persisted 5 months later. Four additional cases of varicella associated with bilateral adrenal hemorrhage have also been reported [9,10,11].

The effects of adrenal gland VZV infection on mineralocorticoid and glucocorticoid release have not been experimentally confirmed. It is also not known whether adrenal dysfunction occurs solely due to direct infection of the adrenal gland or a viral-mediated disruption of upstream signals from the hypothalamus and pituitary gland. Virus-induced loss of infected mineralocorticoid-producing cells within the zona glomerulosa can occur alongside symptoms of tachycardia, hypotension, and shock. A loss of cells within the zona fasciculata can cause symptoms of muscle weakness, lack of energy, low mood, and decreased appetite (as seen in hypocortisolism and Addison’s disease). Loss of cells in the zona glomerulosa can cause erectile dysfunction, reduced bone mass, and reduced sex drive. Loss of chromaffin cells can lead to decreased epinephrine and norepinephrine release, resulting in fatigue, headaches, difficulty sleeping, changes in blood pressure and heart rate, and hypoglycemia. After VZV infection is resolved, and depending on the pace and extent of adrenal regeneration [36], postviral fatigue or “long-hauler” symptoms may persist. Studies on the effects of viruses on adrenal glands and the broader HPA axis are particularly relevant during the current coronavirus disease 2019 (COVID-19) pandemic. Many individuals develop postviral syndrome or long-hauler symptoms that persist for months after contracting the acute disease. A postmortem study showed that 12/28 (46%) of COVID-19 patients had adrenal gland lesions, including necrosis, ischemia, lipid degeneration, hemorrhage, and inflammation [37]. Future studies on the frequency of adrenal involvement during VZV infection and mechanistic studies using adrenal cell cultures in vitro will increase our understanding of the clinical impact of varicella adrenal disease and how viruses impact the adrenals and HPA-axis, guiding potential diagnostics and therapies.

## Figures and Tables

**Table 1:** Histologic and pathologic features of the adrenal gland of two SVV-infected macaques.

	Immunocompetent		Irradiated	
Feature	Cortex	Medulla	Cortex	Medulla
SVV antigen	++	+	++	―
Cowdry A inclusions	+	+	+	―
Necrotic cells	+	―	++	―
PMCs	+	+	+	―
Hemorrhage	+	+	++	++
CD45	+	+	―	―
Tyrosine hydroxylase	―	+	―	+
βIII tubulin	++	―	+	―

Abbreviations: SVV = simian varicella virus; PMC = polymorphonuclear cells.

## Data Availability

The data presented in this study are available on request from the corresponding author.
